# MSC derived EV loaded with miRNA‐22 inhibits the inflammatory response and nerve function recovery after spinal cord injury in rats

**DOI:** 10.1111/jcmm.16965

**Published:** 2021-10-05

**Authors:** Yongjia Sheng, Xiaohong Zhou, Jin Wang, Heping Shen, Shasha Wu, Weiqun Guo, Yi Yang

**Affiliations:** ^1^ Department of pharmacy The Second Affiliated Hospital of Jiaxing University Jiaxing China; ^2^ Department of Ultrasonography The Second Affiliated Hospital of Jiaxing University Jiaxing China

**Keywords:** bone marrow mesenchymal stem cells, EV, miRNA‐22, pyroptosis, spinal cord injury

## Abstract

Our previous research has found that miRNA‐22 can inhibit the occurrence of pyroptosis by targeting GSDMD and decrease the production and release of inflammatory factors. In consideration of the therapeutic effects of mesenchymal stem cells (MSCs), MSCs‐EV were loaded with miRNA‐22 (EV‐miRNA‐22) to investigate the inhibitory effect of EV‐miRNA‐22 on the inflammatory response in SCI in rats in this study. LPS/Nigericin (LPS/NG) was used to induce pyroptosis in rat microglia in vitro. Propidium iodide (PI) staining was performed to observe cell permeability, lactate dehydrogenase (LDH) release assay was adopted to detect cytotoxicity, flow cytometry was conducted to detect pyroptosis level, immunofluorescence (IF) staining was utilized to observe the expression level of GSDMD (a key protein of pyroptosis), Western blot was performed to detect the expression of key proteins. For animal experiments, the T10 spinal cord of rats was clamped by aneurysm clip to construct the SCI model. BBB score, somatosensory evoked potential (SEP) and motor evoked potential (MEP) were performed to detect nerve function. HE staining and Nissl staining were used to detect spinal cord histopathology and nerve cell damage. EV‐miRNA‐22 could inhibit the occurrence of pyroptosis in microglia, suppress the cell membrane pore opening, and inhibit the release of inflammatory factors and the expression of GSDMD. In addition, EV‐miRNA‐22 showed higher pyroptosis‐inhibiting ability than EV. Consequently, EV‐miRNA‐22 could inhibit the nerve function injury after SCI in rats, inhibit the level of inflammatory factors in the tissue and the activation of microglia. In this study, we found that miRNA‐22‐loaded MSCs‐EV (EV‐miRNA‐22) could cooperate with EV to inhibit inflammatory response and nerve function repair after SCI.

## 1 BACKGROUND

Spinal cord injury (SCI) can cause severe dyskinesia and sensory disturbance.^1^ Inflammation caused by inflammatory factors can lead to secondary damage. The mechanism of inflammatory response of SCI is relatively complicated, involving both nerve cell injury and the participation of the immune system.^2^, ^3^ A large number of studies have demonstrated that inflammatory chemokines and cytokines can activate immune cells and central nerve cells in SCI‐triggered inflammatory responses. Microglia are the main immune cells involved in the inflammatory response after SCI and are also the main cells involved in the inflammatory response.^4^, ^5^ Pyroptosis is a novel type of inflammatory death. The process of pyroptosis is mainly mediated by the Caspase family. Among them, Caspase‐1 can mediate the cleavage of Gasdermin D (GSDMD) and pro‐IL‐1β. After cleavage, GSDMD is cleaved into p30‐GSDMD, and p30‐GSDMD forms oligomers after oligomerization and anchors on the cell membrane, causing the formation of cell membrane pores, increasing cell osmotic pressure, causing membrane rupture and the release of a large number of inflammatory factors.^6^, ^7^ Theoretically, the inhibition of GSDMD can suppress pyroptosis, and simultaneously inhibit the activation of microglia and inflammatory response. Our team has previously found that miRNA‐22 is a non‐coding RNA regulated by GSDMD mRNA, which can inhibit neuroinflammatory response and is simultaneously validated in the Alzheimer's disease model.^8^

In recent years, stem cell transplantation has been fully investigated in the treatment of SCI.^9^ Present studies have demonstrated that mesenchymal stem cells (MSCs) can repair the nerve function injury of SCI to a certain extent, promote the regeneration of nerve axons and enhance the repair of motor nerves as well as functional repair.^10^, ^11^ However, the transplantation of MSCs may cause side effects such as tissue immune rejection, teratogenesis, and tumorigenesis, which is simultaneously accompanied with such problems as stem cell survival rate after transplantation.^12^ Therefore, it is urgently needed to exploit a new approach to replace stem cell therapy. EV are vesicle‐like structures secreted by cells. Studies have found that the EV derived from MSCs also exert good effects on the nerve repair of SCI.^13^, ^14^ In addition, MSC‐derived EV greatly attenuate the various side effects caused by MSCs. Therefore, in this study, miRNA‐22 was loaded into the MSC‐derived EV, which was speculated to promote the regeneration of nerve cells and inhibit neuroinflammation, thereby playing a synergistic role.

## 2 MATERIAL AND METHODS

### 2.1 Detection of dynamic expression of miRNA‐22 after SCI in rats

Animal experiments have been reviewed and approved by the Jiaxing University ethics committee. The entire animal experiment conforms to the relevant regulations of animal ethics and welfare, and the whole process conforms to ethical norms.

A total of 36 adult male rats (raised by the Animal Experiment Center of Jiaxing University) were divided into Control, SCI‐12h, SCI‐24h, SCI‐48h, SCI‐72h and SCI‐7D groups (six rats in each group). In brief, the spinal cord was clamped by aneurysm clips to construct the SCI model. The rats were sacrificed after 12 h, 24 h, 48 h, 72 h and 7D, followed by resection of the spinal cord tissue of the T10 segment for examination. To construct the SCI model, rats were fasted for 12 h before the operation, subjected to anaesthesia with 4 mg/kg chloral hydrate, fixed on the plate, and routinely disinfected, followed by 3‐cm longitudinal incision of the connection of the 13th rib and the 13th thoracic vertebrae to expose the skin, fascia and muscles. The T9‐T11 were positioned upwards from the 13 thoracic spinous process, and the T11 spinous process was exposed laterally. After cutting off the supraspinous ligament and interspinous ligament, the Mosquito Clamp was used to remove the T9 and T11, the spinal canal at the T10 spinous process was excised to expose the spinal cord and the aneurysm clamp (clamping force −0.88N) was utilized to clamp the T10 spinal cord for 10 s. The posterior limb of the rats showed no muscle tension, and the spinal cord was congested and swollen, which was sutured after compression to stop bleeding. Rats were maintained in the clean environment and intraperitoneally injected with penicillin once daily for anti‐infection. When rats were sacrificed, the spinal cord was exposed along the original surgical incision, and the T9‐T11 segments were excised and stored at −80°C.

Detection of the expression level of miRNA‐22 by real‐time quantitative PCR (RT‐qPCR): After cutting the pieces, 50–100 mg tissue was added with TRIzol reagent, homogenized in a homogenizer and centrifuged at 12000g at 4°C to collect the supernatant. For 1 ml of TRIzol reagent, 0.2 ml of chloroform was added, mixed well and incubated. After centrifugation at 10,000 *g* at 4°C, the upper supernatant was removed, followed by addition of the same volume of isopropanol. The mixture was mixed well and centrifuged again to acquire RNA. RNA was dissolved in an appropriate amount of RNase water. RevertAid First Strand cDNA Synthesis Kit (Fermentas MBI) was used for reverse transcription of cDNA, followed by detection of miRNA‐22 expression. The primer sequences of miRNA‐22 were as follows: F: 5′‐AAGCTGCCAGTTGAAGAACTGTA‐3′; R: 5′‐GCTGTCAACGATACGCTACGTAAC‐ 3′. The primer sequences of U6 (internal control) were as follows: F: 5′‐CGCTTCGGCAGCACATATA‐3′; R: 5′‐TTCACGAATTTGCGTGTCAT‐3′. The reaction condition was as follows: 37°C for 15min; 85°C for 5 s; 4°C for storage. The 2‐∆∆Ct method was sued to calculate the relative expression of miRNA‐22 accordingly: ∆Ct = −Ct(miRNA‐22)‐Ct(U6), ∆∆Ct = (Ct_target gene_‒Ct_internal control_) experimental group‐(Ct_target gene_‒Ct_internal control_) control group.

### 2.2 Extraction of bone marrow MSCs and their EV and miRNA‐22 loading

Six‐week‐old SD rats were sacrificed by cervical dislocation, followed by isolation of the femur and tibia. After exposing the bone marrow cavity, DMEM (containing FGF2) (Gibco) was used to wash the bone marrow cavity to disperse the cells into single cell suspension. Cells were transferred to the petri dish and the culture medium was changed every three days to discard non‐adherent cells and tissues. MSCs passaging for five times were used for subsequent experiments. miRNA‐22 mimic (Invitrogen) was dissolved in DEPC and transfected into MSCs. In addition, 250 μl of Opti‐MEM and 10 μl of miRNA‐22 mimic were mixed well and incubated for 5 min. The transfection reagent and the plasmid liquid were mixed and incubated for 20 min, the medium was discarded and mixed Opti‐MEME was added to cells for incubation for 72 h. For EV extraction, miRNA‐22 mimic‐transfected MSCs were digested and washed with PBS for three times. DMEM complete medium was prepared with 10% FBS without EV (Gibco). After incubation for 72 h, MDSCs were collected and the EV were isolated by ultra‐high‐speed centrifugation.

1. Observation of EVsome (EV‐miRNA‐22) morphology by electron microscope: A total of 40 ml of culture medium was subjected to ultracentrifugation to isolate EV, and the isolated EV were resuspended in 20–30 μl PBS. Ten microlitre of the sample was aspirated and dropped on the copper mesh for 1 min. The floating liquid was absorbed by the filter paper. Afterwards, 10 μl of 2% uranyl acetate was aspirated and dropped on the copper mesh for 1 min, followed by absorption of the floating liquid by the filter paper and drying for several minutes at room temperature. Finally, the samples were observed and photographed at 80 kv–120 kv.
2. The number and size of the EV were directly tracked by the rate of Brownian motion of EV using the NanoSight NS 300 system (NanoSight Technology, Malvern), configured with a high‐sensitivity sCMOS camera, fast video capture and particle‐tracking software (NanoSight). The samples were diluted 150–3000 times with Dulbecco's PBS (DPBS) without any nanoparticles to attain a concentration of 1–20 × 10^8^ particles per millilitre for analysis. Each sample was measured in triplicate at the camera, which recorded and tracked each visible particle. EVsome numbers and size distribution were explored using the Stokes‐Einstein equation.
3. Detection of EVsome markers: NP‐40 (Beyotime) was used extract the protein, and the supernatant was subjected to protein quantitation by the BCA kit (Beyotime). The protein sample was mixed with 5× loading buffer to a final volume of 20 μl, After boiling for 8 min, the protein sample was subjected to electrophoresis at 80 V and further 120 V, transferred to the PVDF membrane (300 mA constant current for 0.5–2 h), blocked with 5% skimmed milk powder for 2 h, incubated with proper primary monoclonal antibodies (CD63, CD81 and ALIX) diluted in TBST, washed with TBST twice and incubated with horseradish peroxidase‐labelled goat anti‐rabbit secondary antibody (Abcam, USA), and finally subjected to visualization by ECL method. Image Pro‐Plus 6.0 software was used for optical density analysis.
4. Detection of the level of miRNA‐22 loading by RT‐qPCR: The expression of miRNA‐22 in EV was detected accordingly.

### 2.3 The effects of EV‐miRNA‐22 on pyroptosis in microglia of rats

Rat microglia (MG) (Procell) were cultured with RPMI‐1640 (Procell). When cells reached to 70% confluency, cells were divided into Control group, LPS/NG group, EV group and EV‐miRNA‐22 group. Cells of LPS/NG group were pre‐treated with 1 μg/ml lipopolysaccharide (LPS) (Sigma, USA) for 5 h, followed by addition of 10 μM Nigericin (MCE, Shanghai, China) to induce pyroptosis. Cells of EV and EV‐miRNA‐22 groups were pretreated with EV and EV‐miRNA‐22 for 4 h, followed by LPS/NG treatment to induce pyroptosis.

1. Detection of cytotoxicity lactate dehydrogenase (LDH) assay: LDH kit (Solarbio) was purchased to detect cytotoxicity. After Nigericin treatment to induce pyroptosis, the LDH release rate after 2 h.
2. Detection of propidium iodide (PI) absorption rate: PI absorption rate was assessed every 20 min within 2 h after Nigericin intervention (a total of six detections). In brief, cells were treated with 1 μg/ml PI, 120 nM NaCl, 5 mM glucose, 1.5 mM CaCl2, 1 mM magnesium chloride and 0.1% bovine serum albumin (BSA). Cells were detected for absorbance at a wavelength of 533/617 nm. PI absorption rate (%) = (OD_Sample_‒OD_background_)/(OD_maximum_‒OD_background_).
3. Detection of cell membrane pore size by EtBr/EthD2 staining: Two types of red non‐permeable membrane dyes with different molecular weights were selected, namely, micromolecular dye EtBr (molecular weight of 394 Da, Sigma, US) and macromolecular dye EthD2 (molecular weight of 1293 Da, Sigma, US). Microglia were inoculated into 24‐well plates, added with 0.1% Triton X‐100 to for 10 min before intervention, washed with PBS for 3 times (5 min each); stained with EtBr or EthD2 for 5–10 min, rinsed with PBS and added anti‐fluorescence quencher for mounting, followed by observation and photography under fluorescence microscope. The experiment was repeated for three times.
4. Immunofluorescence (IF) staining for GSDMD: Microglia were inoculated into a 6‐well plate, treated accordingly, fixed with freshly prepared 4% paraformaldehyde (PFA) for 10 min, washed with PBS for three times, permeabilized with 0.2% Triton X‐100 for 10 min, blocked with 2% BSA for 30 min, incubated with monoclonal antibody against GSDMD (Abcam, Massachusetts, USA, dilution 1:300) at room temperature for 1 h, washed with PBS for three times, added with IgG antibody (Abcam) for labelling, counterstained with 0.5 μg/ml DAPI staining reagent (Solarbio), washing with PBS twice and mounted, followed by observation under fluorescence microscope.
5. Detection of apoptotic level by Annexin‐FITC/PI via flow cytometry: After LPS/NG treatment, cells were collected, gently resuspended in PBS and counted. The resuspended cells were centrifuged at 200 *g* for 5 min. After discarding the supernatant, 195 μl Annexin V‐FITC‐binding solution was added to gently resuspend cells, followed by addition of 5 μl Annexin V‐FITC and gentle mixture. After incubation in dark at room temperature for 10 min, samples were subjected to flow cytometry for detection.
6. Detection of relative protein expression by Western blot: The protein expression of GSDMD and p30‐GSDMD was detected accordingly (Abcam).
7. Detection of the expression of inflammatory factors in the culture medium by ELISA: After Nigericin treatment, cells were collected, centrifuged at 3,000 *g* to collect the supernatant to detect the levels of inflammatory factors (IL‐1β, IL‐18 and TNF‐α) by using ELISA kits (Nanjing Jiancheng Biotechnology Co., Ltd.). After the reagents were added and incubated according to the manufacturer's instructions, the absorbance value was detected at 450 nm by a microplate reader (BioTek) and the results were shown as pg/ml.

### 2.4 The effects of EV‐miRNA‐22 on SCI in rats

A total of 32 rats were divided into sham operation (Sham), SCI, SCI + EV, SCI + EV‐miRNA‐22 groups. 0.5 ml EV or EV‐miRNA‐22 solution (100 μg EV, 200 μg/ml) was injected to rats of SCI + EV or SCI + EV‐miRNA‐22 group by the tail vein one day before model construction. SCI model was constructed according to the above protocol. Afterwards, EV or EV‐miRNA‐22 was injected into the tail vein every three days. Rats of Sham and SCI groups were injected with saline for 15 continuous days.

1. The nerve function of rats was evaluated by BBB method, which was assessed every 1–3–9–15 days after drug administration.
2. Somatosensory evoked potential (SEP) assay: Rats were anaesthetized with 2% pentobarbital with disappeared corneal reflex and even breath. The anaesthetized rats were placed on the operating floor. After drilling rat's skull to expose the right cerebral sensory cortex, the electrode was inserted under the frontal skin to expose the sciatic nerve, followed by electrical stimulation and subsequent analysis for 100 ms. The latency of SEP N1 wave was recorded and the electrophysiological recovery was cautiously observed.
3. Motor evoked potential (MEP) assay: After drug administration, the nerve conduction waveform and the latency and amplitude changes of N1 wave were evaluated every 1–3–9–15 days (the operation method was consistent with SEP assay).
4. Haematoxylin and eosin staining: Paraffin‐embedded spinal cord tissue was serially cut into 6 μm‐thick sections, and one section of each brain tissue was subjected to Haematoxylin and eosin staining to determine the ischemic marginal area. (1) Dewaxing: xylene (I) 2.5 min, xylene (II) 2.5 min, absolute ethanol 2 min, 95% ethanol 1 min, 85% ethanol 1 min, 75% ethanol 1 min, tap water 2 min, distilled water 2 min; (2) Staining: staining with haematoxylin for 5 min, rinsing with tap water for 1 min, incubating with 75% hydrochloric acid and ethanol for 30 s, washing with tap water, soaking in warm water (50°C) for 5 min, washing with distilled water for 1 min, 95% ethanol for 1 min, adding acidified eosin ethanol for 1.2 min; (3) dehydration, transparent and mounting: 95% ethanol (I) 1min, 95% ethanol (II) 1min, absolute ethanol (I) 1min, absolute ethanol (II) 1min, xylene carbolic acid (3:1) 1min, xylene (I) 1 min, xylene (II) 1 min, mounting with neutral gum. The nucleus showed blue‐black and the cytoplasm showed light red.
5. Nissl staining: After paraffin embedding and ethanol treatment, the spinal cord tissue was stained with Nissl staining solution at 37–50°C for 8–10 min, washed with distilled water twice and 95% ethanol once, treated with xylene for 5 min for transparent and mounted with neutral resin, followed by observation.
6. Detection of cytokine expression by ELISA: After sacrifice, the injured spinal cord tissue was resected from rats, homogenized in liquid nitrogen, lysed with NP‐40, centrifuged to collect the supernatant, followed by detection of cytokine levels by ELISA kit according to the manufacturer's instruction.

### 2.5 Statistical analysis

All measurement data were expressed as $\bar{\chi }$±s, and SPSS 17.0 was used for analysis and data processing. After the homogeneity of variance test, two independent sample *t*‐test was used for comparison between the two groups. One‐way ANOVA was used for comparison among three groups, followed by LSD method for subsequent pairwise comparisons between the groups. Two‐sided *p *< 0.05 indicated statistical significance.

## 3 RESULTS

### 3.1 The expression of miRNA‐22 in SCI model and the identification of EVsome

**FIGURE 1:**
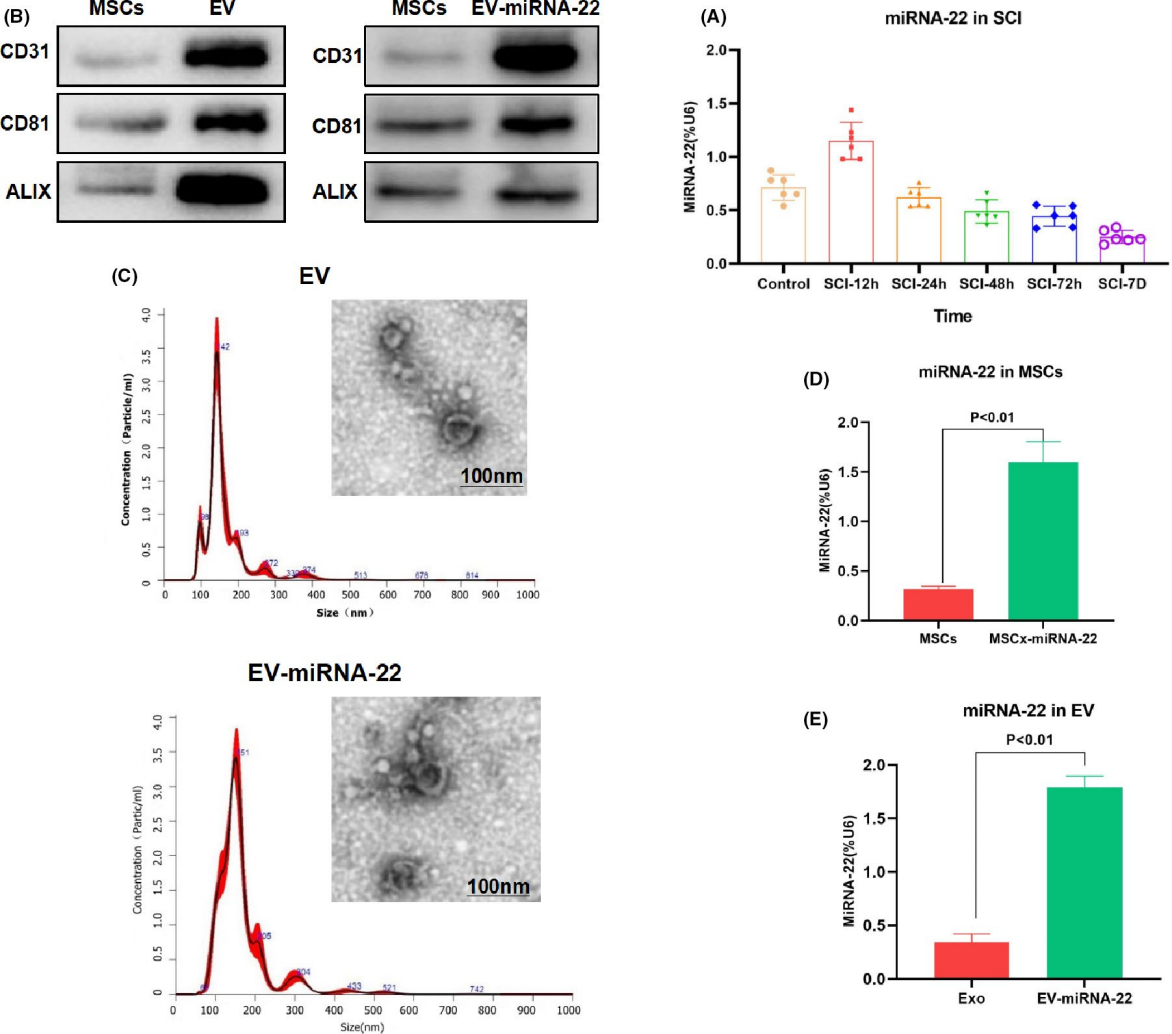
The dynamic changes of miRNA‐22 and the characterization of EV. (A) The expression of miRNA‐22 was first increased and then decreased after the occurrence of SCI. The expression of miRNA‐22 was up‐regulated in the acute phase, and the expression of miRNA‐22 was significantly lower than that of Control group after 48 h. (B) The expression of EVsome markers (CD63, CD81 and ALIX) was relatively high in EV and EV‐miRNA‐22, which was significantly higher than that of MSCs. (C) EV and EV‐miRNA‐22 showed pie‐like shapes, which were in line with the appearance characteristics of EV. In addition, the particle size analysis showed that EV and EV‐miRNA‐22 were approximately 100 nm in size, indicating that miRNA‐22 did not affect EVsome formation. (D) After miRNA‐22 transfection in MSCs, the expression of miRNA‐22 was significantly up‐regulated. Comparison between groups, *p *< 0.05. (E) The expression of miRNA‐22 in EV‐miRNA‐22 was significantly higher than that of EV. Comparison between groups, *p *< 0.05

The expression of miRNA‐22 was detected on the 7D after SCI in rats. As a result, the expression of miRNA‐22 in the spinal cord tissue of rats were increased during the acute phase (12 h) and later down‐regulated. The expression of miRNA‐22 was significantly lower than Control group after 48 h (Figure 1A). EV and EV‐miRNA‐22 were characterized and found that miRNA‐22‐loaded MSC‐derived EV had a pie‐like shape similar to EV, which was consistent with the appearance of EV. In addition, the particle size analysis showed that EV and EV‐miRNA‐22 were both at 100 nm, indicating that miRNA‐22 did not affect the stability of EV (Figure 1C). When detecting EVsome markers, we found that the expression of CD63, CD81 and ALIX was significantly higher in EV and EV‐miRNA‐22 than MSCs (Figure 1B). After miRNA‐22 overexpression in MSCs, the level of miRNA‐22 was significantly increased (Figure 1D). And the level of miRNA‐22 in EV‐miRNA‐22 was significantly higher than that of EV (Figure 1E).

### 3.2 The effects of EV‐miRNA‐22 on pyroptosis in microglia

**FIGURE 2:**
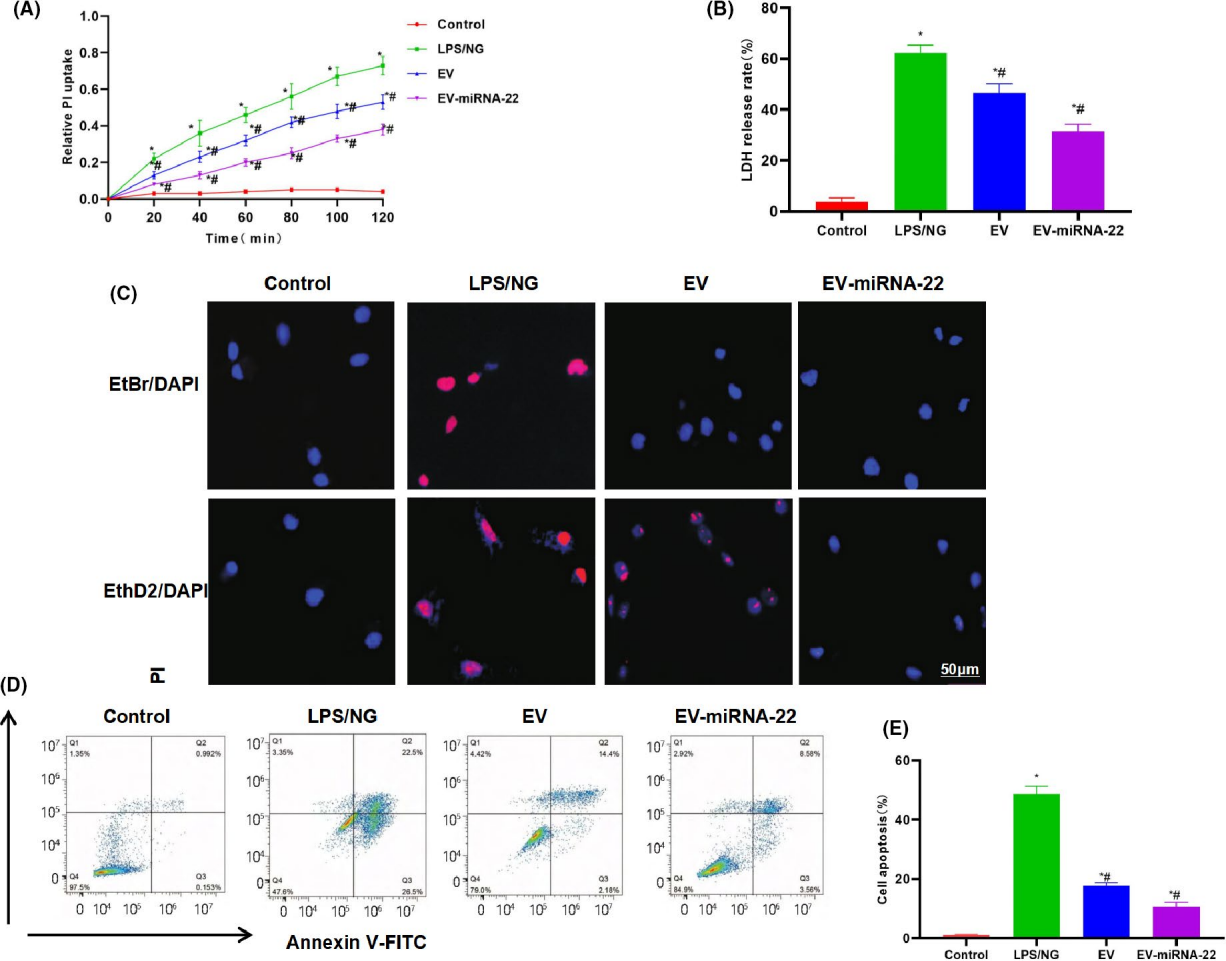
The effects of EV‐miRNA‐22 on pyroptosis in microglia. (A) The PI uptake rate showed that PI was not significantly uptaken in cells of Control group, the PI uptake rate was significantly increased in LPS+NG group and the PI uptake rate was decreased in EV and EV‐miRNA‐22 groups. Moreover, the PI uptake rate was significantly lower in EV‐miRNA‐22 group than that of EV group. Comparison with Control group, *p < 0.05; comparison with LPS + NG group, ^#^ *p *< 0.05. (B) LDH release rate showed that LPS/NG could increase LDH release, indicating obvious cell injury. While EV and EV‐miRNA‐22 could inhibit the release of LDH. And the release rate of EV‐miRNA‐22 was significantly lower than that of EV. Comparison with Control group, *p < 0.05; comparison with LPS + NG group, ^#^ *p *< 0.05. (C) EtBr/EthD2 staining. EtBr/EthD2 and EtBr/EthD2 could enter into cells of LPS/NG groups. While EV‐miRNA‐22 could inhibit EtBr/EthD2 entry and EV could inhibit EtBr/EthD2 entry to a certain extent. (D and E) Detection of pyroptosis. EV‐miRNA‐22 could inhibit the occurrence of pyroptosis and decrease the proportion of pyroptosis. Compared with Control group, *p < 0.05; comparison with LPS + NG group, ^#^ *p *< 0.05

The relative PI uptake assay could detect the opening degree of cell membrane pores. As a result, the relative uptake rate of PI was low in Control group, without significant increase over time, indicating the low opening level of cell membrane pores. In LPS + NG group, the opening level of cell membrane was significantly enhanced and the PI uptake rate was increased; while the PI uptake rate was decreased in EV and EV‐miRNA‐22 groups, which was significantly lower than that in LPS + NG group (Figure 2A). When detecting LDH release rate, we found that the LDH release rate was significantly higher in LPS + NG group than that of Control group, and the inhibition level of LDH release rate was significantly higher in EV‐miRNA‐22 group than that of EV group (Figure 2B). EtBr is a macromolecular dye, while EthD2 is a micromolecular dye. Both EtBr and EthD2 could enter into cells in LPS + NG group, which had a high opening level of membrane pores. While EV‐miRNA‐22 could inhibit the opening of cell membrane pores, thereby attenuating the entrance of EtBr and When EthD2 into cells. EV could also inhibit the opening of cell membrane pores, but not as potent as EV‐miRNA‐22 (Figure 2C). According to the results of flow cytometry, EV‐miRNA‐22 could inhibit pyroptosis, with significantly lower pyroptotic rate than EV group (Figure 2D,E).

**FIGURE 3:**
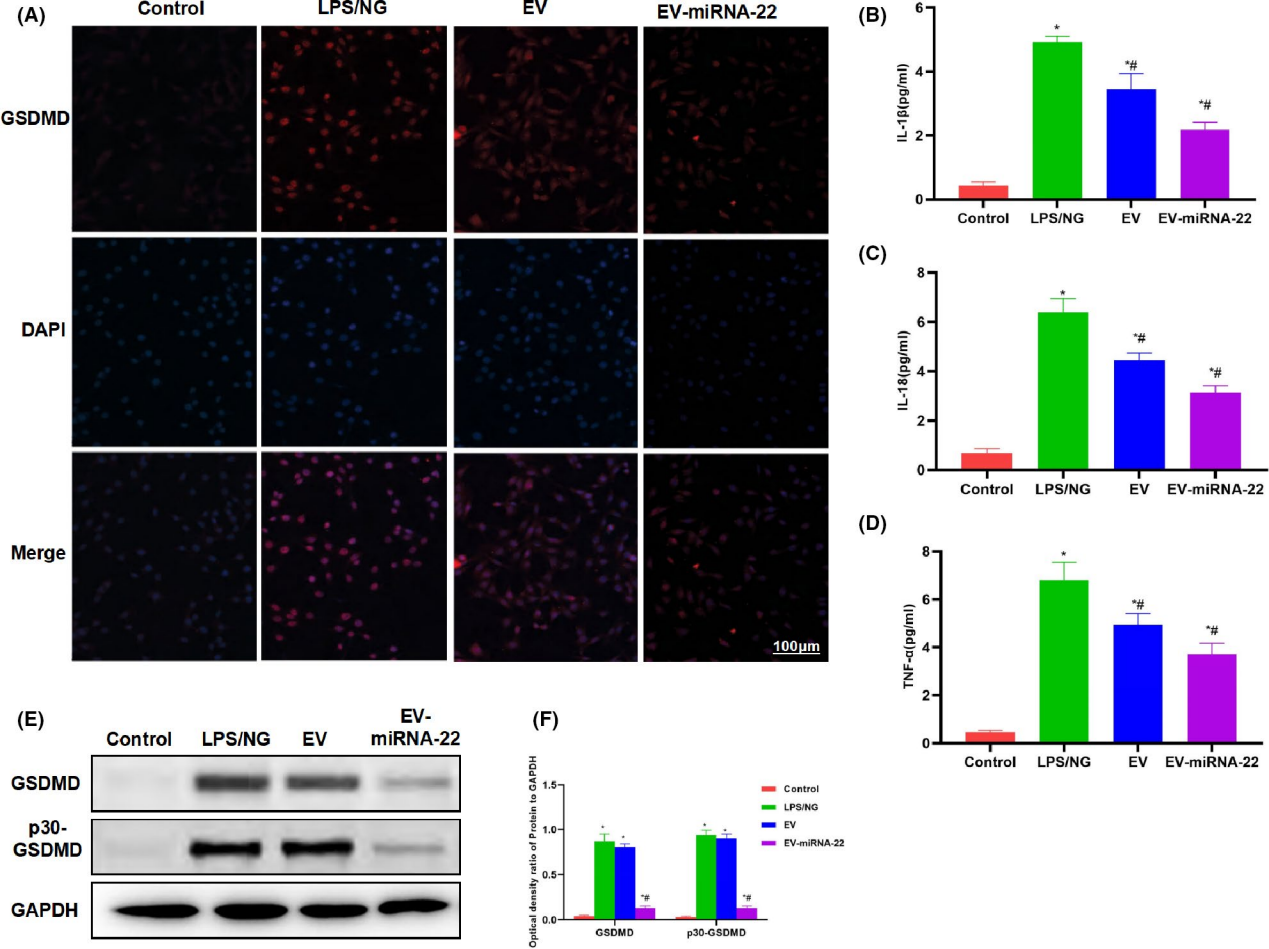
The effects of EV‐miRNA‐22 on the levels of GSDMD and inflammatory factors. (A) IF staining of GSDMD. LPS/NG promoted the expression of GSDMD, while EV and EV‐miRNA‐22 could inhibit the expression of GSDMD, with significantly decreased fluorescence intensity. (B–D) Detection of inflammatory factor expression. The expression of inflammatory factors (IL‐1β, IL‐18 and TNF‐α) was significantly up‐regulated in pyroptosis, which was higher than that of Control group. EV and EV‐miRNA‐22 could inhibit the release of inflammatory factors. In particular, the level of inflammatory factors was lower in EV‐miRNA‐22 group than that in EV group. Comparison with Control group, *p < 0.05; comparison with LPS + NG group, ^#^ *p *< 0.05. (E and F) Protein detection revealed that EV‐miRNA‐22 could inhibit the expression of GSDMD and p30‐GSDMD. Comparison with Control group, *p < 0.05; comparison with LPS + NG group, ^#^ *p *< 0.05

For mechanism study, we found that EV and EV‐miRNA‐22 could inhibit the expression of GSDMD. IF staining showed that EV‐miRNA‐22 had a stronger ability to inhibit the expression of GSDMD than EV (Figure 3A). When detecting inflammatory factors, EV‐miRNA‐22 could better inhibit the occurrence of pyroptosis and the release of inflammatory factors, which was significantly different than LPS/NG group and was significantly lower than EV group (Figure 3B–D). The detection of GSDMD and p30‐GSDMD revealed that EV‐miRNA‐22 could inhibit the expression of GSDMD and decrease the levels of GSDMD and p30‐GSDMD (Figure 3E,F).

### 3.3 The effects of EV‐miRNA‐22 on the nerve function of SCI rats

The BBB score of SCI, EV group and EV‐miRNA‐22 group was 0. Rats were unable to move with posterior limbs and could only crawl with anterior limbs. After post‐operative intervention of EV and EV‐miRNA‐22, the neurological function of rats was recovered to different degrees, which was significantly different from SCI model (*p *< 0.05). The neurological recovery level of rats in EV‐miRNA‐22 group was significantly higher than that of EV group, suggesting that EV‐miRNA‐22 could better promote the nerve function recovery in rats. Results were shown in Table 1.

**TABLE 1:** BBB score(mean ± SD, Score)

Group	1 d	3 d	9 d	15 d
Sham	21.0 ± 0.0	21.0 ± 0.0	21.0 ± 0.0	21.0 ± 0.0
SCI	0^*^	0.56 ± 0.2^*^	1.05 ± 0.22^*^	3.44 ± 0.88^*^
SCI + EV	0^*^	1.04 ± 0.43^*#^	4.55 ± 0.43^*#^	6.98 ± 0.89^*#^
SCI + EV‐miRNA‐22	0^*^	1.88 ± 0.56^*#^	6.44 ± 0.87^*#^	8.14 ± 0.44^*#^

Note

Compared with Sham group, ^*^ *p *< 0.05;Compared with SCI group, ^#^ *p *< 0.05.

Somatosensory evoked potential assay revealed that the evoked potential waveform of rats in Sham group was normal, and the evoked potential waveform disappeared after SCI. After EV and EV‐miRNA‐22 intervention, the SEP value of rats was significantly changed from SCI (*p *< 0.05). The conduction function of posterior limbs of rats were significantly recovered. And the improvement was more obvious in EV‐miRNA‐22 group, which was significantly different from EV group. The results were shown in Table 2.

**TABLE 2:** Comparison of Latency Time of N1 Waveform in SEP Experiment (mean ± SD, s)

Group	1 d	3 d	9 d	15 d
Sham	12.47 ± 2.11	13.12 ± 1.87	12.76 ± 2.44	13.66 ± 2.44
SCI	35.44 ± 3.65^*^	34.65 ± 4.65^*^	32.54 ± 3.98^*^	30.98 ± 4.87^*^
SCI+EV	36.65 ± 5.33^*^	32.54 ± 6.44^*#^	29.98 ± 4.88^*#^	26.98 ± 6.12^*#^
SCI+EV‐miRNA‐22	35.12 ± 4.14^*^	30.44 ± 4.55^*#^	27.87 ± 3.98^*#^	22.55 ± 4.19^*#^

Note

Compared with Sham group, ^*^ *p *< 0.05;Compared with SCI group, ^#^ *p *< 0.05.

After constructing SCI model, the N1 latency was significantly higher than that in Sham group. After EV and EV‐miRNA‐22 intervention, the N1 latency was significantly shortened than SCI model (*p *< 0.05). The conduction function of posterior limbs of rats was significantly recovered. And the improvement in EV‐miRNA‐22 group was more obvious, which was significantly different from EV group. The results were shown in Table 3.

**TABLE 3:** Latent time comparison of N1 waveform in MEP experiment (mean ± SD, s)

Group	1 d	3 d	9 d	15 d
Sham	4.12 ± 0.54	4.55 ± 0.65	4.09 ± 0.55	4.28 ± 0.87
SCI	30.87 ± 3.55^*^	31.98 ± 1.54^*^	28.87 ± 2.12^*^	26.98 ± 3.54^*^
SCI+EV	31.87 ± 3.51^*^	29.88 ± 2.43^*#^	26.98 ± 3.54^*#^	24.88 ± 4.33^*#^
SCI+EV‐miRNA‐22	30.55 ± 3.54^*^	27.87 ± 3.55^*#^	24.54 ± 3.55^*#^	22.98 ± 3.12^*#^

Note

Compared with Sham group, ^*^ *p *< 0.05; Compared with SCI group, ^#^ *p *< 0.05.

### 3.4 The effects and mechanisms of EV‐miRNA‐22 on SCI rats

**FIGURE 4:**
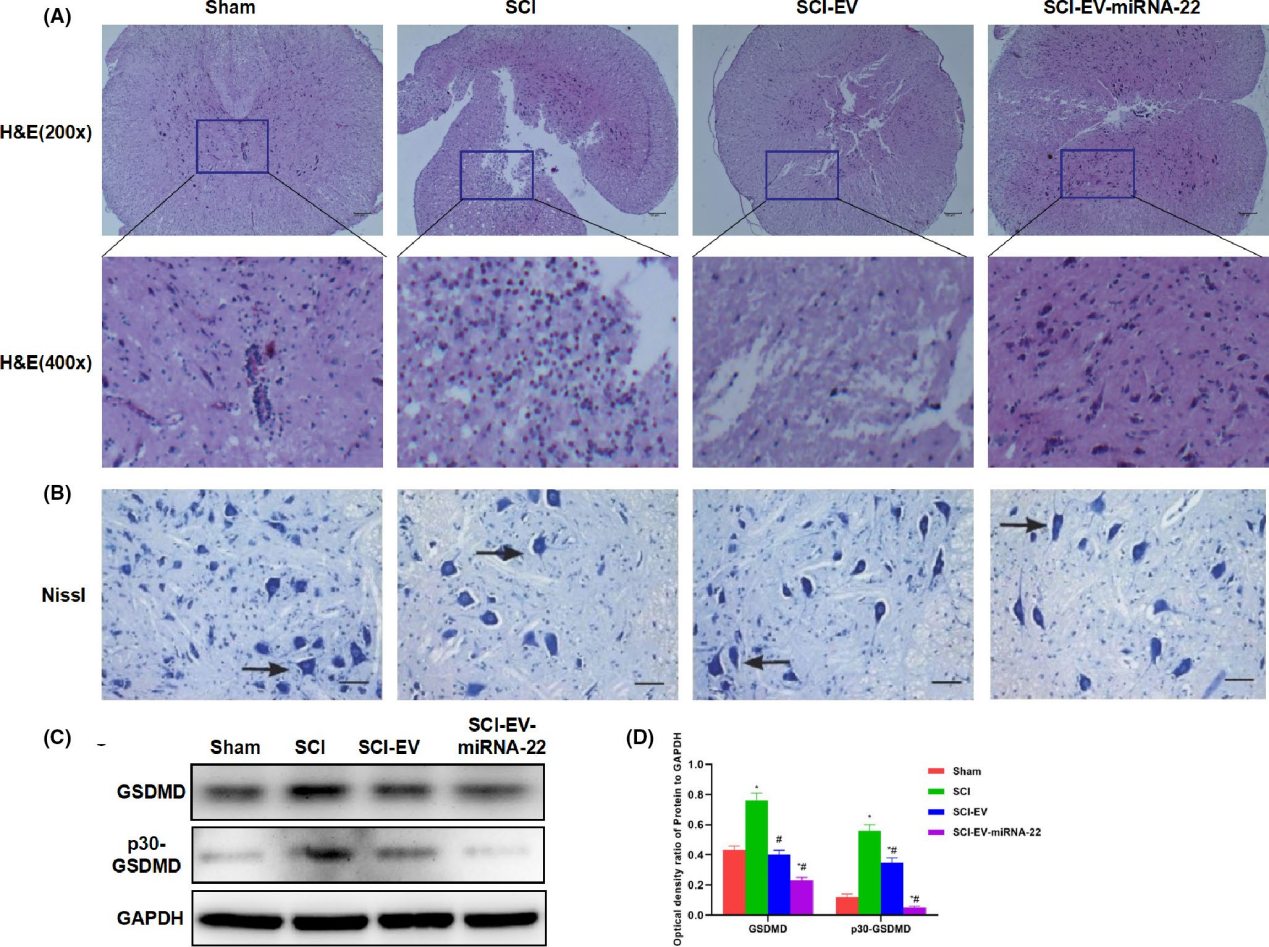
The effect of EV‐miRNA‐22 on the pathology of SCI rats. (A) Haematoxylin and eosin staining of spinal cord tissue in rats. Cells in the spinal cord were severely damaged in rats of SCI model, with obvious oedema and inflammation. Cell injury was significantly attenuated and inflammatory response was decreased in EV and EV‐miRNA‐22 groups. The scar tissue and inflammatory response in EV‐miRNA‐22 group was milder than EV group. (B) Nissl staining. Cell injury was obvious in SCI, with sparse positive cells, which was significantly different from Sham group. Cell injury was significantly relieved in EV and EV‐miRNA‐22 groups. (C and D) The expression of GSDMD and p30‐GSDMD was significantly up‐regulated in SCI than that of Sham group; while the expression of GSDMD and p30‐GSDMD was significantly decreased in EV and EV‐miRNA‐22 groups than that in SCI group

Haematoxylin and eosin staining showed no inflammation or oedema, no obvious damage to the tissue in the spinal cord tissue of Sham rats. In SCI model, the spinal cord cells were severely damaged, with obvious oedema and inflammation. Cell injury and inflammation were significantly attenuated in EV and EV‐miRNA‐22 groups, which was significantly different from the model group. The tissue damage was further relieved and cell damage was alleviated in EV‐miRNA‐22 group (Figure 4A). Nissl staining showed dense cells and high positive rate after staining the nerve cells of Sham rats. After SCI, the cell injury was obvious and the positive cells were sparse, which was significantly different from Sham group; while the cell injury was significantly alleviated in EV and EV‐miRNA‐22 groups (Figure 4B). GSDMD detection showed that the expression of GSDMD and p30‐GSDMD was significantly up‐regulated in SCI, which was significantly higher than that of Sham; while EV and EV‐miRNA‐22 could inhibit the expression of GSDMD and p30‐GSDMD (Figure 4C,D).

**FIGURE 5:**
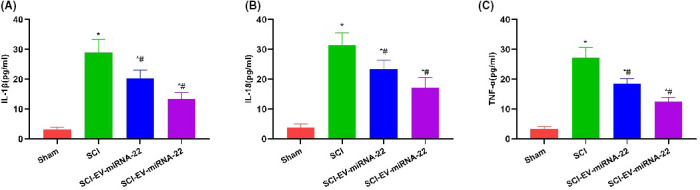
Test results of pyrolysis and inflammatory factor levels in rat spinal cord microglia. (A–C) Results of inflammatory factor detection. The expression of inflammatory factors (IL‐1β, IL‐18 and TNF‐α) in spinal cord was significantly up‐regulated in SCI. EV and EV‐miRNA‐22 could inhibit the release of inflammatory factors; in particular, the level of inflammatory factors in EV‐miRNA‐22 was lower than that in EV group. Comparison with Sham group, *p < 0.05; comparison with SCI group, ^#^ *p *< 0.05

The detection of inflammatory factors also revealed that EV and EV‐miRNA‐22 could inhibit the expression of inflammatory factors in the spinal cord, and EV‐miRNA‐22 had better inhibitory effects than EV (Figure 5A–C).

## 4 DISCUSSION

Spinal cord injury is one of the common causes of disability in young people.^13^ The pathological process of SCI includes two processes: namely, primary injury and secondary injury. Primary injury is defined as the direct damage of SCI by external force, causing the rupture of nerve cell membrane, thereby leading to irreversible nerve cell necrosis.^14^, ^15^ For secondary damage, at this stage, cell contents are rapidly released after cell necrosis,^16^ such as glutamate, reactive oxygen species (ROS), potassium ions and cathepsin B (CTSB).^17^, ^18^, ^19^ The leakage of these substances can cause inflammation of the nervous tissue and even lead to cell death. Cell death includes multiple types, such as autophagic death, programmed necrosis and pyroptosis; however, it is not yet clear which type plays a predominant role.^20^, ^21^ Present studies show that pyroptosis might play a relatively important role. Pyroptosis is a newly discovered programmed cell death, accompanied by the production of various inflammatory mediators, including IL‐1β and IL‐18.^22^ There are many cell types in the spinal cord, including neurons, microglia and astrocytes.^23^ After SCI, neurons, astrocytes and microglia can generate neurotoxic molecules, such as ROS and CTSB.^24^ Inflammasomes can be formed by activation of these molecules. Previous studies have demonstrated that different types of inflammasomes are expressed differentially in neurons, astrocytes and microglia, indicating that these cells might response differently to these activation signal.^6^, ^25^ Microglia have been found as the main source of NLRP3 inflammasome expression in rat SCI model.^26^ The cleavage of GSDMD by activated caspase‐1 is also a key step in the occurrence of pyroptosis. Recent studies have found that cleavage occurs at the FLTD peptide site of GSDMD,^27^ and Ac‐FLTD‐CMK blocks the cleavage of GSDMD by the FLTD peptide site. Studies have also found that Necrosulfonamide (NSA) could interact with the cleaved GSDMD, inhibit the oligomerization of P30‐GSDMD and prevent the formation of pores, thereby preventing pyroptosis in nerve cells.^28^ The main source of inflammatory damage in SCI is the activation of microglia. More studies have also found that microglia, as a special type of macrophages, will express a large amount of inflammatory factors in the case of pyroptosis, which is the essence of the inflammatory response. Inhibition of scorch death in SCI has also become a key means to inhibit inflammation.

Our team has previously found that miRNA‐22 is a regulatory non‐coding RNA of GSDMD, and miRNA‐22 has been confirmed to inhibit the activation and pyroptosis of microglia by inhibiting GSDMD.^8^ Stem cells (MSCs) can inhibit the inflammatory response and cell damage of SCI, promote the regeneration of nerve axons and blood vessels,^28^ attenuate the formation of astroglial scars and tissue cavities, and enhance motor nerve repair and functional repair. However, stem cells have multi‐directional differentiation functions.^29^ The suppression of MSCs may cause side effects such as tissue immune rejection, teratogenesis and tumorigenesis,^30^ and simultaneously cause such problems as stem cell survival after transplantation.^31^ Numerous studies have found that transplantation of MSC‐derived EV can repair nerve damage and function recovery after SCI,^32^ which can also avoid certain unknown adverse reactions caused by MSCs. In this study, we first detected the dynamic changes of miRNA‐22 in SCI. As a result, the expression of miRNA‐22 was significantly up‐regulated in the acute phase, and later showed low expression over time, which was significantly lower than that of Control group. We further loaded miRNA‐22 with MSCs‐EV. Consequently, miRNA‐22 transfection into MSCs significantly increased the expression of miRNA‐22 in EV. Meanwhile, the EV in EV and EV‐miRNA‐22 were morphologically normal and uniformly distributed in particle size, therefore, miRNA‐22 loading had no significant effect on EV. In rat microglia, we found that EV‐miRNA‐22 could inhibit LPS/NG‐induced pyroptosis. Although EV also had the same effect, the effect was weaker than EV‐miRNA‐22. EV‐miRNA‐22 could significantly inhibit the opening level of cell membrane pores, and the dye cannot enter into cells. Meanwhile, it inhibited the release of inflammatory factors and the expression of GSDMD and p30‐GSDMD, which was consistent with expectations. EtBr/EthD2 is a macromolecular/micromolecular dye,^33^ and the opening of cell membrane pores can promote a large amount of dye influx. However, EV‐miRNA‐22 perfectly inhibited this phenomenon. When detecting inflammatory factors, EV‐miRNA‐22 could also inhibit the secretion of inflammatory factors.

In the SCI model constructed by the clipping method, SCI rats showed severe inflammatory response, significantly up‐regulated secretion of inflammatory factors, obvious necrosis, inflammatory response and scar in tissue. Nissl staining showed that severe injury of nerve cells in SCI. EV and EV‐miRNA‐22 could well inhibit tissue damage after SCI, and simultaneously down‐regulate the expression of inflammatory factors. Of note, the effect of EV‐miRNA‐22 was superior to that of EV group. The BBB score and electrophysiological test also showed that EV and EV‐miRNA‐22 could repair the nerve function of rats and partially restore nerve function.

## 5 CONCLUSION

In this study, we have found that miRNA‐22 can inhibit the pyroptosis of microglia. The combination of MSCs‐EV and miRNA‐22 can further inhibit the neuroinflammatory response after SCI, thereby improving the neurological function after SCI in rats. EV‐miRNA −22 shows good therapeutic potentials for SCI mainly by cooperating with MSCs‐EV.

## CONFLICT OF INTEREST

No Competing interests.

## AUTHOR CONTRIBUTIONS

**Yongjia Sheng:** Investigation (equal); Methodology (equal); Project administration (equal). **Xiaohong Zhou:** Data curation (equal); Resources (equal); Visualization (equal). **Jin Wang:** Conceptualization (equal); Methodology (equal); Project administration (equal); Resources (equal); Writing‐original draft (equal). **Heping Shen:** Formal analysis (equal); Methodology (equal); Software (equal); Validation (equal); Writing‐original draft (equal). **Shasha Wu:** Data curation (equal); Project administration (equal); Resources (equal); Supervision (equal). **Weiqun Guo:** Formal analysis (equal); Visualization (equal); Writing‐review & editing (equal). **Yi Yang:** Formal analysis (equal); Resources (equal); Writing‐review & editing (equal).

## ETHICAL APPROVAL AND CONSENT TO PARTICIPATE

The study was approved by Ethics Committee.

## CONSENT FOR PUBLICATION

This article was published with the approval of all the authors.

## Data Availability

The data that support the findings of this study are available from the corresponding author upon reasonable request.
